# Arteria lusoria associée à un tronc bi carotidien: à propos d'un cas et revue de la littérature

**DOI:** 10.11604/pamj.2015.22.376.8430

**Published:** 2015-12-16

**Authors:** Safae Khnaba, Meryem Menany, Mouna Moukinebillah, Touriya Amil, Bouchayb Radouane

**Affiliations:** 1 Service d'Imagerie Médicale, Hôpital Militaire d'Instruction Mohamed V, Rabat, Maroc

**Keywords:** Arc aortique, arteria lusoria, tronc bicarotidien, aortic arch, arteria lusoria, bicarotid trunk

## Abstract

L'arteria lusoria ou artère sous Clavière droite retro-œsophagienne constitue la malformation de l'arc aortique la plus fréquente, elle peut être associée à d'autres anomalies congénitales du cœur et des gros vaisseaux, notamment le tronc bi carotidien qui constitue un tronc commun donnant naissance aux deux artères carotides primitives internes. Nous rapportons le cas d'une patiente chez qui on a diagnostiqué un adénocarcinome gastrique, le scanner thoraco-abdominopelvien réalisé dans le cadre de son bilan d'extension a permis la découverte fortuite d'une artère aberrante retro-œsophagienne associé à un tronc bi carotidien.

## Introduction

La crosse aortique et ses branches peuvent être le siège de variations anatomiques, L'anomalie la plus commune concerne l'artère sous clavière droite qui prend son origine directement de l'aorte et rejoindre ainsi le membre supérieur droit en empruntant un trajet aberrant, son incidence est de l'ordre de 0.5 à 2% dans la population générale, et peut être associée dans 30% des cas à un tronc bicarotidien [1]. Elle est le plus souvent asymptomatique et de découverte fortuite.

## Patient et observation

**Figure 1:**
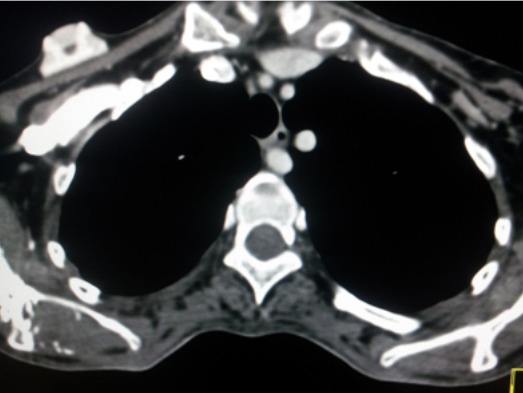
Coupe axiale en fenêtre médiastinale après injection de produit de contraste: artère lusoric retro-œsophagienne (flèche)

**Figure 2:**
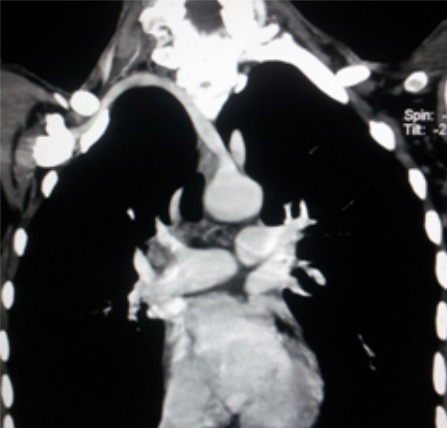
Reconstruction coronale en MIP, Naissance directe de l'artère sous Clavière droite à partir de l'aorte (flèche)

**Figure 3:**
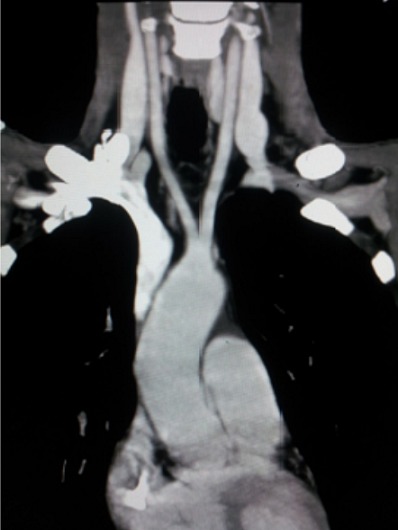
Reconstruction sagittale en MIP, Naissance directe de l'artère sous Clavière droite à partir de l'aorte qui empreinte un trajet retro-œsophagien (étoile)

Il s'agit d'une patiente âgée de 45ans, sans antécédents particuliers notables, qui a présenté il y a 6 mois des épi gastralgies intermittentes avec pyrosis, elle a été mise sous traitement symptomatique. L’évolution a été marquée par l'aggravation des épi gastralgies avec apparition de vomissements et de méléna avec syndrome anémique d'aggravation progressive. Une fibroscopie a été réalisée et a objectivé un processus tumoral ulcéro-bourgeonnant au niveau du corps gastrique. L'analyse anatomopathologique des prélèvements biopsiques a confirmé le diagnostic d'adénocarcinome gastrique moyennement différencié. Elle a été adressée dans notre service de radiologie pour faire un scanner thoraco-abdominopelvien dans le cadre du bilan d'extension. L'analyse des coupes thoraciques en fenêtre médiastinale après injection de produit de contraste iodé a mis en évidence la naissance d'un vaisseau de la crosse de l'aorte, qui croise la ligne médiane, chemine en retro-‘sophagien et enfin se dirige en avant et à droite pour aller au niveau de la région axillaire droite (Figure 1,Figure 2). Les 2 artères carotides primitives prenaient naissance à partir d'un tronc commun court naissant directement de la crosse de l'aorte (Figure 3).

## Discussion

L'artère sous Clavière droite aberrante ou arteria lusoria constitue la malformation de l'arc aortique la plus fréquente, son incidence est de l'ordre de 0.5 à 2% dans la population générale [1]. Cette anomalie vasculaire a été initialement décrite, à notre connaissance, en 1735 par Hunauld, [2]. Et c'est en 1794 que Bayford a décrit les signes cliniques de cette anomalie vasculaire sous le terme de dysphagie lusoria [3]. Elle est le résultat d'une aplasie du 4^ème^ arc aortique droit, compensée par une persistance de l'aorte dorsale droite ipsilatérale, la persistance de cette dernière, qui dans l’état physiologique régresse permet de corriger l'agénésie d'un des 4 arcs aortiques. La physiopathologie de cette malformation demeure inconnue et pourrait comporter un facteur d'origine hémodynamique en complément du « fond » génétique et évolutif [4]. La variation anatomique la plus fréquente qui s'associe avec l'artéria lusoria est la naissance de l'artère carotide primitive gauche du tronc artériel brachiocéphalique qui prend alors le nom de truncus bicaroticus; ca se voit dans 30% des cas, comme le cas de notre observation [5]. Sur la plan clinique, l'arteria lusoria est souvent asymptomatique, vu que cette dernière ne forme pas d'anneau complet autour de l’œsophage ni de la trachée, elle est découverte dans la majorité des cas de façon fortuite lors d'une exploration thoracique pour d'autres pathologies. Elle devient symptomatique essentiellement dans trois cas: d'une part quand l’œsophage et la trachée sont comprimés entre l'arteria lusoria en arrière et le tronc bicarotidien en avant [6], d'autres part lorsqu'il existe un anévrysme de cette artère qui constitue une complication redoutable, et enfin avec l’âge lors d'une dégénérescence athéroscléreuse de l'artère, ou de la survenue d'une dysplasie fibro-musculaire [7]. Le principal signe clinique est la dysphagie appelé dysphagie lusoria, il s'agit d'une dysphagie aux solides, une dyspnée ou une toux chronique peuvent se voir témoignant d'une compression de la trachée. Sur la radiographie standard, cette artère se manifeste par une opacité de tonalité hydrique médiastinale supérieure, en contact avec le bord supérieur droit de la crosse de l'aorte. Une opacification de l’œsophage permet de mettre en évidence une empreinte oblique en haut et à droite sur la paroi œsophagienne. Ce transit œsophagien est surtout réalisé chez le nourrisson, plus rarement chez l'adulte. La naissance de cette artère est mieux visualisé par les moyens d'imagerie en coupe notamment le scanner et l'angio-IRM. Aucune étude n'a été réalisée dans le sens de l’étude de la sensibilité, spécificité et supériorité d'un examen par rapport à un autre dans l'exploration de cette malformation [8]. En scanner, il s'agit d'un vaisseau qui nait de la face postérieure de l'aorte, présente un trajet retro-œsophagien pour remonter en haut et en avant dans la région axillaire. L'IRM, si réalisée montre facilement cette anomalie, en coupe coronale postérieure, elle met en évidence un vaisseau prenant naissance de l'isthme de l'aorte, qui a un trajet oblique en haut et à droite et sui rejoint la région axillaire, les coupes sagittales montrent la localisation rétro-œsophagienne [8]. Aucun traitement n'est indiqué pour une arteria lusoria asymptomatique. Un traitement n'est justifié que si elle entraine une dysphagie gênante ou en cas de complication de cette artère à savoir les anévrysmes quels soient symptomatique ou pas.

## Conclusion

L'arteria lusoria est une malformation vasculaire rare souvent asymptomatique de découverte fortuite. Son diagnostic doit amener le radiologue à rechercher dans anomalies cardiaques et des gros vaisseaux. L'association à un tronc bi carotidien est l'anomalie la plus fréquente.
